# Longitudinal association of retinal morphology and white matter progression in retinal vasculopathy with cerebral leukoencephalopathy and systemic manifestations

**DOI:** 10.3389/fneur.2025.1724411

**Published:** 2025-12-08

**Authors:** Henok Getahun, Rajendra S. Apte, Wilson X. Wang, Vivian Chen, Mae Gordon, Julia Huecker, John P. Atkinson, M. Kathryn Liszewski, Slim Fellah, Serguei V. Astafiev, Jonathan J. Miner, Andria L. Ford

**Affiliations:** 1 John F. Hardesty Department of Ophthalmology and Visual Sciences, Washington University School of Medicine, St. Louis, MO, United States; 2 Department of Neurology, Washington University School of Medicine, St. Louis, MO, United States; 3 Center for Biostatistics and Data Science, Washington University School of Medicine, St. Louis, MO, United States; 4 Department of Medicine, Division of Rheumatology, Washington University School of Medicine, St. Louis, MO, United States; 5 Mallinckrodt Institute of Radiology, Washington University School of Medicine, St. Louis, MO, United States; 6 Division of Rheumatology, Department of Medicine, University of Pennsylvania Perelman School of Medicine, Philadelphia, PA, United States

**Keywords:** imaging, retina, biomarkers, vasculopathy, leukoencephalopathy

## Abstract

**Background:**

Retinal vasculopathy with cerebral leukoencephalopathy and systemic manifestations (RVCL-S) is a rare small vessel disease marked by vision loss and neurocognitive deterioration. We sought to characterize the relatedness of the underlying retinal structure and cerebral disease.

**Methods:**

Participants with RVCL-S underwent optical coherence tomography (OCT) and brain MRI at baseline and 2 years. Total macular volume (TMV) and central subfield thickness (CST) were generated from OCT images. Neuroimaging metrics of ischemic brain injury included white matter hyperintensity (WMH) volume, white matter microstructure (mean diffusivity), and normalized white matter volume. Associations between retinal and neuroimaging metrics were assessed.

**Results:**

Eleven RVCL-S participants were included. Reduction in TMV was associated with reduced normalized white matter volume (*β* = 0.021, 95% CI [0.006, 0.036], *p* = 0.0137), increased WMH volume (*β* = −11.0, 95% CI [−17.4, −4.0], *p* = 0.0046), and a near-significant increase in mean diffusivity (*β* = −0.017, 95% CI [−0.035, −0.001], *p* = 0.057). CST was not associated with neuroimaging metrics. Percent change in TMV was associated with percent change in mean diffusivity (*β* = −0.61, 95% CI [−1.14, −0.084], *p* = 0.028).

**Conclusion:**

A metric of retinal structure, TMV, may provide a marker of cerebral disease severity in RVCL-S. Additional studies are needed to demonstrate whether early measures of TMV predict cerebral disease progression.

## Introduction

Retinal vasculopathy with cerebral leukoencephalopathy and systemic manifestations (RVCL-S) is a rare small vessel disease caused by frameshift mutations in the gene encoding the three-prime repair exonuclease (TREX1), a 3′ to 5′ DNA specific exonuclease (1). Clinical features of RVCL-S include progressive vision loss, neurocognitive deterioration, hepatopathy, nephropathy, and other systemic manifestations leading to premature death typically within 5–10 years of symptom onset (2, 3).

Neuroimaging manifestations in RVCL-S patients demonstrate white matter hyperintensities (WMH) on T2-weighted Fluid-Attenuated Inversion Recovery (FLAIR) consisting of punctate lesions with and without nodular enhancement and periventricular mass lesions with surrounding edema (3). Retinal vasculopathy due to RVCL-S has been characterized using multimodal structural and functional measurements, revealing enlargement of the foveal avascular zone and peripheral retinal nonperfusion (4, 5). These changes in retinal perfusion are accompanied by gross retinal findings characteristic of RVCL-S, including macular edema with vascular leakage, telangiectasias, microaneurysms, and micro-infarcts (3–6).

Recently, we conducted a clinical trial in a cohort of participants with RVCL-S which examined longitudinal changes in retinal structure including measurements of central subfield thickness (CST), defined as the retinal thickness in a 1 mm diameter ring centered around the fovea, and total macular volume (TMV), representing the retinal volume in a 6 mm diameter ring centered around the fovea (5, 6). It was previously found that TMV was decreased in patients with RVCL-S compared to healthy controls, however, whether retinal morphology is temporally related to neuroimaging progression in RVCL-S has not been investigated (6, 7). In Alzheimer’s disease, Huntington’s disease, and sporadic cerebral small vessel disease (cSVD), changes in the neurosensory retina such as ganglion cell-inner plexiform layer thinning and enlargement of the foveal avascular zone have been associated with alterations in brain imaging and cognitive decline (8–11). This study’s primary aim was to examine the temporal association between retinal morphology and cerebral progression in a monogenic cSVD. We hypothesized that metrics of retinal structural integrity and morphology, specifically TMV and CST, and their change over time, would be associated with neuroimaging metrics of cSVD (5, 7). If true, these data would support future investigation into retinal biomarkers of cerebral disease progression in both monogenic and sporadic forms of cSVD.

## Materials and methods

### Study participants

Patients with genetic testing-confirmed RVCL-S were enrolled in this study between January 2021 and December 2021 at Washington University in St. Louis School of Medicine. The current report is a post-hoc analysis of data from a previously published clinical trial (Crizanlizumab for the Treatment of Retinal Vasculopathy with Cerebral Leukoencephalopathy) (5). Inclusion criteria were: age greater than 25 and a genetic testing-confirmed diagnosis of RVCL-S. The exclusion criteria were: contraindications to MRI, acute infection, Human Immunodeficiency Virus positivity, untreated latent tuberculosis, active hepatitis B or C, active herpes zoster, pregnancy or breastfeeding status, known hypersensitivity to any study agent, a white blood cell count below 4 × 10^9^/L, an absolute neutrophil count below 1.5 × 10^9^/L, a platelet count below 100 × 10^9^/L, liver function test values greater than three times the upper limit of normal within 30 days of enrollment, treatment with monoclonal antibodies within 30 days of enrollment, treatment with anticoagulation within 30 days of study enrollment, and treatment with any investigational drugs within 14 days of enrollment. All participants gave informed consent for participation in the clinical trial. Study participants underwent optical coherence tomography (OCT) to obtain CST and TMV values as well as brain imaging with MRI.

### Study design

Using imaging data collected for a prospective, single-arm RVCL-S clinical trial, we performed a secondary analysis to examine the relationship between retinal and cerebral pathology across the baseline and 2-year timepoints from the RVCL-S study population.

### Brain image protocol and processing

All participants underwent magnetic resonance imaging via Siemens 3 T scanner (Siemens Healthineers, Erlangen, Germany) with a 64-channel head coil. T1 weighted Magnetization-Prepared-Rapid-Gradient-Echo (MPRAGE); echo time (TE)/repetition time (TR) = 2.95/1,800 ms, inversion time (TI) = 1,000 ms, flip angle = 8 degrees, resolution = 1.0 × 1.0 × 1.0 mm^3^, and 2D FLAIR (TE/TR = 93/9,000 ms, TI = 2,500 ms, resolution = 0.86 × 0.86 × 3 mm^3^) images were produced. MPRAGE images were segmented into gray and white matter using Statistical Parametric Mapping version 12 (SPM12, Wellcome Institute of Neurology, London, UK). FMRIB Software Library’s (FSL, Oxford University, UK) Integrated Registration and Segmentation Tool (FIRST) was used to segment subcortical gray matter (12, 13). FMRIB’s Linear (rigid) Image Registration Tool (FLIRT) was used to register images obtained within a scan session. The volume of white matter hyperintensities (WMH) was segmented from FLAIR images using automated segmentation with manual correction confirmed by a vascular neurologist (A.F) who was blinded to participant ID (5, 14). Diffusion-weighted imaging (TE/TR = 89/10,100 ms, isotropic 2.0 × 2.0 × 2.0 mm^3^; *b* = 0–1400 s/mm^2^ [FOV = 224 mm]) was also conducted to assess white matter microstructure as defined by white matter mean diffusivity (MD) which was quantified using the FSL Diffusion Toolbox (FDT, Oxford University, UK) (15). The normalized white matter volume was quantified by dividing the white matter volume by total intracranial volume to account for variation in head size (16).

#### Cognitive testing

Participants underwent cognitive assessment at the same timepoints as when they underwent neuroimaging. The Digit Symbol Substitution Test (DSST), a reliable test of working memory and processing speed, was administered to participants. A Category Fluency test in which participants were given 2 min to name items in a semantic category was also administered. Finally, participants underwent a Montral Cognitive Assessment (MoCA). Scores on these measures of cognitive performance were recorded, with higher scores denoting better performance.

### Retinal optical coherence tomography imaging

Optical coherence tomography (OCT) images of the optic disc and macula were obtained using the Heidelberg Retina Angiography plus OCT Spectralis system (5). Automated retinal segmentation was used to generate thickness and volume measurements for each segment of the Early Treatment of Diabetic Retinopathy Study (ETDRS) macular map (5, 17, 18). Volumes from each segment of the ETDRS map were added to obtain TMV values, measured in mm^3^. CST values were documented by recording the average thickness values of the central segment of the ETDRS map, measured in μm.

### Statistical analysis

Statistical analysis was conducted using R studio version 2024.09.0 and SAS version V9.4. Associations between retinal and neurologic imaging metrics were evaluated using repeated measures mixed models. Data from one eye from each participant at each timepoint was used as a fixed effect in each model. The eye chosen for each participant corresponded to what authors considered, the “worst eye,” defined as the eye with the lowest TMV and CST values at each timepoint. Time, denoting data from baseline or 2-years, was also added as a fixed effect in each model. Additionally, each model incorporated a random intercept to account for inter-time and inter-eye correlations. Analysis was performed both by including all data and by excluding outliers. Outliers were defined as any value above or below two standard deviations from the mean for each variable. Differences between worst-eye TMV and CST values, neuroimaging endpoints at end of study and at baseline, and cognitive scores at end of study and baseline were assessed using Wilcoxon signed-rank test. Finally, associations between rate of change in TMV and CST and rate of change of neurologic variables were also assessed. Rate of change for retinal variables were quantified by averaging values from the left and right eyes at each timepoint; the difference between averaged values at the final and baseline timepoint were calculated and subsequently divided by the averaged baseline value to determine the percent change. Rate of change of neurologic variables was determined using the same process but without the need to average values given each participant had one neurologic measurement at each timepoint. Linear regression modelled the percent change of TMV and CST as predictors of percent change in the three neuroimaging endpoints. For all analyses, a *p*-value below 0.05 was considered statistically significant.

## Results

### Participants

Eleven patients (22 eyes) with genetic testing-confirmed RVCL-S were included. Of the 11 participants, one participant experienced cystoid macular edema in the left eye, resulting in high outlier values for TMV and CST (18.43 mm^3^ and 891 μm at 2-year timepoint, respectively) which were excluded from analysis. Demographic information is reported in Table 1.

**Table 1:** Cohort characteristics.

Characteristic	
Sex, count (%)	
Male	3 (27)
Female	8 (73)
Age (years)^a^	46.6 (7.8)
Race, count (%)	
White	10 (90.9)
Asian	1 (0.9)
TMV (mm^3^)^b^	8.08 (0.48)
CST (μm)^b^	257.6 (24.5)
WMH (mL)^c^	9.16 (8.66)
MD (mm^2^/s)^c^	0.69 (0.014)
Normalized white matter volume at baseline^c^	0.30 (0.018)

a Mean age at baseline (SD).

b Mean retinal values at baseline (SD) are taken from eyes considered the worst eye for each metric, defined as the eye (left or right) with the lower TMV and CST value.

c Mean metrics of cerebral white matter disease (SD) were quantified from baseline values. White matter volume was normalized by dividing by total intracranial volume.

Table depicting the demographic information of the study cohort and mean values of retinal and neurologic metrics at baseline.

SD, standard deviation; TMV, total macular volume; CST, central subfield thickness; WMH, white matter hyperintensity; MD, mean diffusivity.

### Progression in metrics of retinal morphology, cerebral structure, and cognitive function

For retinal structural metrics, the median TMV values (IQR) at baseline and 2 years were 8.3 mm^3^ (7.64, 8.44) and 7.9 mm^3^ (7.4, 8.3), respectively, declining significantly (*p* = 0.005). The median CST values (IQR) at baseline and end of study did not differ and were 257 μm (236.5, 282.5) and 249 μm (230.5, 282.5), respectively (*p* = 0.44).

Neuroimaging endpoints of cSVD included WMH volume, MD, and normalized white matter volume. The median WMH volume (IQR) at baseline and end of study were 5.9 mL (3.1, 13.3) and 8.2 mL (6.0, 20.7), respectively, increasing significantly over 2 years (*p* = 0.002). The median MD values (IQR) at baseline and end of study were 0.696 (0.688, 0.70) and 0.704 (0.695, 0.718), respectively, increasing over the course of 2 years (*p* = 0.05). Median normalized white matter volumes (IQR) at baseline and end of study were 0.296 (0.29, 0.31) and 0.287 (0.277, 0.311), respectively, decreasing over the course of the study (*p* = 0.009).

Cognitive testing included DSST, category fluency, and MoCA. The median DSST scores (IQR) at baseline and end of study were 43 (38, 48) and 42 (35.5, 48), respectively (*p* = 0.28). The median category fluency scores (IQR) at baseline and end of study were 34 (30.5, 39.5) and 33 (30, 38), respectively (*p* = 0.24). Finally, the median MoCA scores (IQR) at baseline and end of study were 28 (25.5, 29) and 27 (24, 28.5), respectively (*p* = 0.69).

### Longitudinal associations of retinal structure with neuroimaging endpoints of cSVD

**Figure 1:**
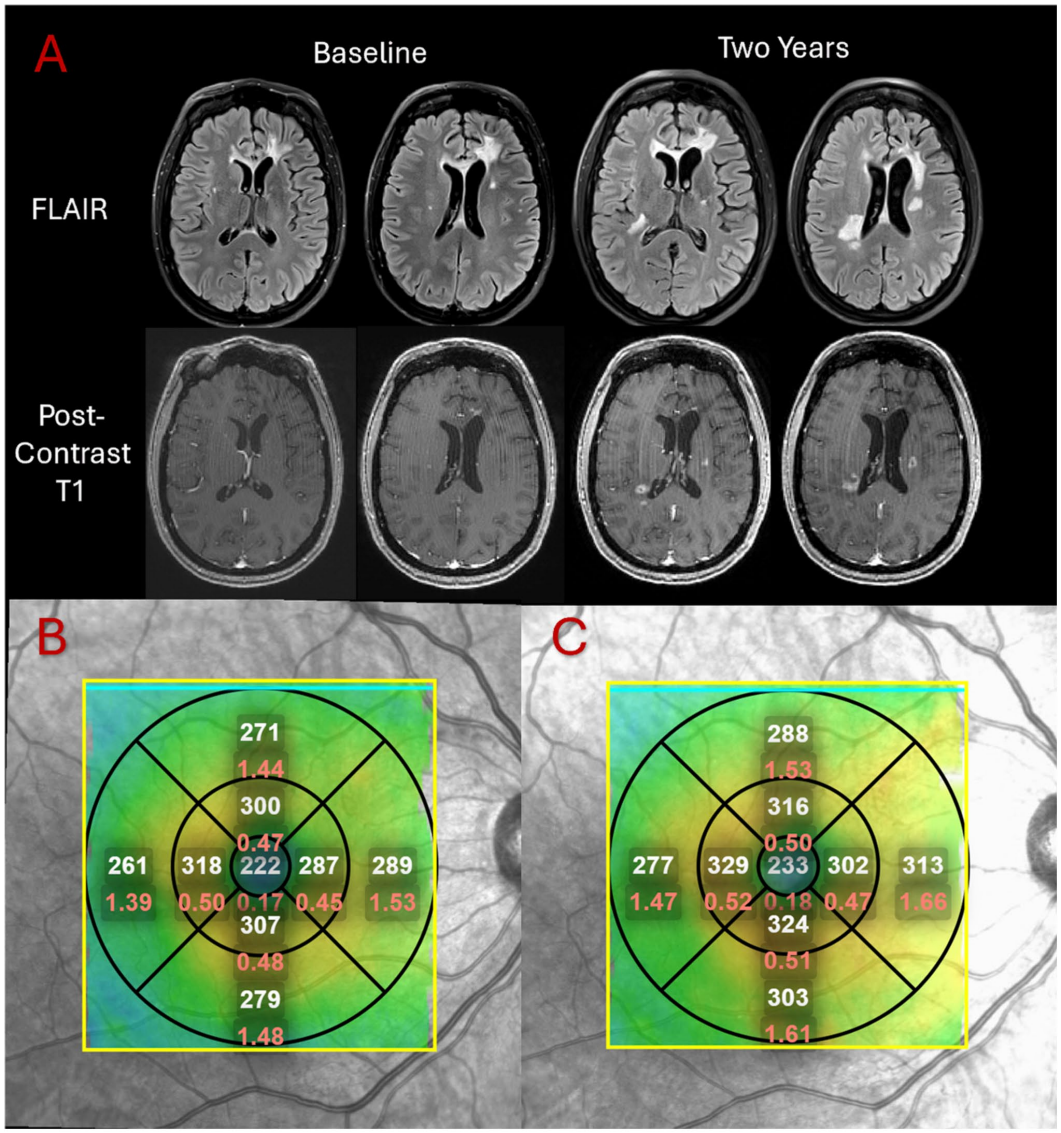
Example of macular volume, central subfield thickness, and white matter hyperintensities in a single participant. **(A)** Figure depicting FLAIR and post-contrast T1-weighted brain images from one participant at baseline and 2-year follow-up. Extension of existing lesions and new lesions can be noted. **(B)** Figure depicting an optical coherence tomography image of the macula in the same participant at baseline. The Early Treatment of Diabetic Retinopathy Study (ETDRS) grid is centered around the fovea, with average thickness values of each segment presented in white and volume measures in mm^3^ presented in red. **(C)** Figure depicting a macular optical coherence tomography image of the same participant at end-of-study showing lower TMV values at follow-up, quantified by adding volume measurements in all segments.

Associations between retinal measurements from the worst eye and neurologic measures of cSVD are summarized in Table 2. One outlier in WMH volume was identified corresponding to a high value of 43.6 mL. When this outlier was excluded, the association between TMV and WMH volume was significant (*β* = −11.0, 95% CI [−17.4, −4.0], *p* = 0.0046). Reduced TMV had a near significant association with elevated MD (*β* = −0.017, 95% CI [−0.035, −0.001], *p* = 0.057) and was significantly associated with reduced normalized white matter volume (*β* = 0.021, 95% CI [0.006, 0.036], *p* = 0.0137). CST was not significantly associated with WMH volume, MD, or normalized white matter volume (*p* = 0.57, 0.70, and 0.76, respectively). Figure 1 depicts a visual representation of one participant’s TMV, CST, and WMH volume on FLAIR.

**Table 2:** Associations between total macular volume, central subfield thickness, and neuroimaging metrics.

Neurologic variable	Regression coefficient (SE)	95% CI	*p*-value
Dependent variable = TMV (mm^3^)			
WMH volume (mL)	−5.3 (4.6)	−14.3 to 3.7	0.27
Mean diffusivity (mm^2^/s)	−0.017 (0.008)	−0.035 to −0.001	0.057
Normalized white matter volume	0.021 (0.0078)	0.006–0.036	0.0137
Dependent variable = CST (μm)			
WMH volume (mL)	0.057 (0.098)	−0.13 to 0.25	0.57
Mean diffusivity (mm^2^/s)	−0.00007 (0.00017)	−0.0004 to 0.0002	0.70
Normalized white matter volume^a^	0.00006 (0.0002)	−0.0003 to 0.0004	0.76

a White matter volume was normalized by dividing by total intracranial volume.

Table depicting associations between retinal and neurologic metrics as assessed by repeated measures mixed models.

CI, confidence interval; SE, standard error; TMV, total macular volume; CST, central subfield thickness; WMH, white matter hyperintensity.

### Percent change in retinal and neuroimaging metrics of cSVD

**Figure 2:**
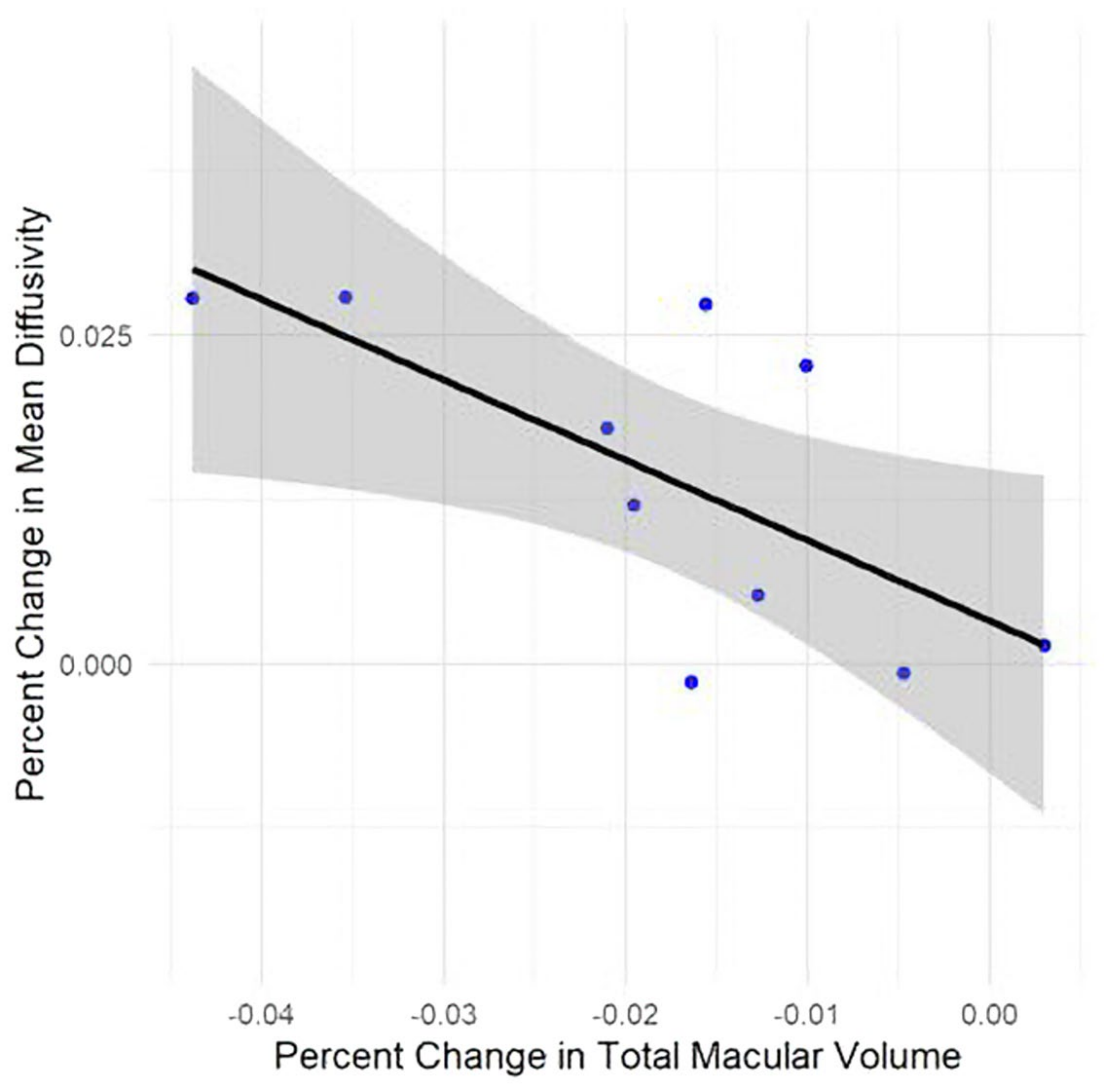
Line graph with overlaid datapoints depicting the relationship between percent change in total macular volume and percent change in white matter mean diffusivity (regression coefficient: −0.61, 95% CI [−1.14, −0.084], *R*^2^: 0.47, *p* = 0.028). Shaded area depicts the 95% confidence interval of the linear regression model used to generate the plot. An increase in mean diffusivity indicates decreasing white matter microstructural integrity.

The rate of change in TMV was negatively associated with the rate of change in MD (*β* = −0.61, 95% CI [−1.14, −0.084], *R*^2^: 0.47, *p* = 0.028, Figure 2). Remaining associations between the rate of change of TMV and CST and the rate of change in neurologic metrics are summarized in Supplementary Table 1.

## Discussion

RVCL-S is an ultimately fatal small vessel disease that, among many systemic effects, causes reduced visual acuity and neurocognitive impairment. Although the effect of disease on the retina has been established using both qualitative and quantitative methods, associations between these retinal changes and neurologic changes have not been quantitatively examined (4, 5). In other neurodegenerative and cerebral small vessel diseases, research has illustrated associations between morphologic changes in the retina and neurologic disease (9–11, 19). Given the increasing identification of retinal correlates of cerebral disease, we sought to evaluate the associations between TMV, CST, and neurologic disease markers in participants with RVCL-S.

Since patients with RVCL-S have reduced macular volumes and retinal thinning, we hypothesized that decreasing TMV and CST would be associated with increased WMH lesion volume, reduced white matter microstructural integrity, and white matter atrophy as these measures increase with disease severity (7, 16). Anatomic retinal changes may be due to progressive retinal ischemia and resultant cell death, although the exact mechanism of these changes in RVCL-S are yet to be elucidated. This theory was, in part, substantiated by the significantly lower TMV value at the end of study as compared to baseline, although the same was not true for CST, suggesting the reduction of TMV in RVCL-S is progressive. Results also supported the hypothesis that reduction in TMV is associated with white matter atrophy such that reduced macular volume in RVCL-S may progress in an analogous manner to white matter degeneration. Additionally, TMV was significantly associated with WMH volume after removal of an outlier. This may suggest that reduction in TMV progresses in parallel with increasing cerebral disease activity and severity. Finally, the association between TMV and MD, as a metric of microstructural integrity likely due to progressive small vessel ischemia, was borderline significant, potentially due to limited sample size, although further study is needed to substantiate this. Notably, the association between percent change in retinal TMV and white matter MD supports the notion that retinal and cerebral microstructural degeneration are occurring simultaneously and are proportional to one another. The shared embryologic origin of the retina and brain as well as their vasculature may underpin the associations described, similar to the mirroring of retinal vascular changes and other cerebral vascular diseases (20, 21). In contrast to TMV, CST was not associated with neurologic endpoints of cSVD. The central most aspect of the macula, the fovea, is avascular and largely relies on choroidal rather than retinal circulation for oxygen (22). This may be why central subfield thickness is less sensitive to superficial and deep capillary plexus ischemia from RVCL-S compared to extrafoveal regions (23). This is supported by the stability in CST over the course of our study.

Interestingly, cognitive test scores did not significantly decline over the course of 2 years in our cohort. This may be due to the relatively short follow up time and the inclusion of patients at various stages of disease, with some experiencing cognitive decline and others remaining relatively stable. Compounded by a small sample size, these factors likely influenced the lack of statistical significance.

There are study limitations. As RVCL-S is a rare disease, only 11 participants with complete brain and eye datasets were available, resulting in relatively low statistical power and high chance of type 2 error. Additionally, the small sample size precluded the inclusion of potentially meaningful covariates during analysis such as age, sex, and diagnosis of hypertension. These factors and others are likely to influence both retinal morphology and cerebral white matter disease burden. Furthermore, correction for multiple comparisons was not performed as this study was exploratory in nature, with the purpose of informing future studies. Confirmatory studies are needed to further investigate associations before establishing clinical and research utility of potential retinal biomarkers. Finally, our study was a secondary analysis of data from a clinical trial evaluating crizanlizumab (5). This may hamper generalizability and affect the nature of the associations found or undetected (5).

Taken together, our results suggest that TMV may serve as a retinal metric for stratifying patients by cerebral disease severity and reflecting rate of cerebral progression in patients with RVCL-S. These data support further investigation of TMV as a tool to measure efficacy of treatments in future clinical trials. Additionally, TMV may be an especially promising biomarker of cSVD given the relative ease, cost-effectiveness, and reproducibility of OCT imaging (20, 24). Our findings add to the mounting evidence that cSVDs, both symptomatically and silently, impact the retina, and changes in retinal morphology serve as a window to cerebral pathology and disease progression.

## Data Availability

The original contributions presented in the study are included in the article/Supplementary material, further inquiries can be directed to the corresponding author.
